# External female genitalia of *Triatoma jatai*, *Triatoma costalimai* and *Triatoma williami* (Hemiptera: Reduviidae: Triatominae)

**DOI:** 10.1186/s13071-020-04418-2

**Published:** 2020-10-29

**Authors:** Simone Caldas Teves, Teresa Cristina Monte Gonçalves, Simone Patrícia Carneiro de Freitas, Catarina Macedo Lopes, Ana Laura Carbajal-de-la-Fuente, Jacenir Reis dos Santos-Mallet

**Affiliations:** 1 Laboratório Interdisciplinar de Vigilância Entomológica em Diptera e Hemiptera, Instituto Oswaldo Cruz - Fiocruz, Rio de Janeiro, Brasil; 2 Fundação Oswaldo Cruz Piauí, Teresina, Piauí Brasil; 3 Centro Nacional de Diagnóstico e Investigación en Endemo-Epidemias (CeNDIE), Administración Nacional de Laboratorios e Institutos de Salud “Dr. Carlos Malbrán” (ANLIS), Ciudad Autónoma de Buenos Aires, Argentina; 4 Consejo Nacional de Investigaciones Científicas y Técnicas (CONICET), Ciudad Autónoma de Buenos Aires, Argentina; 5 Departamento de Ciências Biológicas, Universidade Iguaçu (UNIG), Nova Iguaçu, Rio de Janeiro, Brasil

**Keywords:** Triatomines, Taxonomy, Genitalia, Scanning electron microscopy

## Abstract

**Background:**

Taxonomic identification of triatomines is generally performed based on aspects of their external morphology. However, the use of a multidisciplinary approach, considering morphological aspects of the external genitalia, morphometry, genetics, and phylogeography has been suggested, especially for similar and/or cryptic species. The rupestral species *Triatoma jatai* Gonçalves et al., 2013, *Triatoma costalimai* Verano & Galvão, 1959 and *Triatoma williami* Galvão et al., 1965, which are morphologically similar, have been found naturally infected with *Trypanosoma cruzi* (Chagas, 1909) in wild, peridomestic, and intradomestic environments, representing a risk of new outbreaks of Chagas disease. This study presents morphological description complementation of these species, with an emphasis on the structures of the female external genitalia, using scanning electron microscopy.

**Methods:**

The females of *T. jatai* and *T. costalimai* (*n* = 10 of each) were captured in the Brazilian municipalities of Paranã and Aurora do Tocantins and were identified with the use of a dichotomous key for the Matogrossensis subcomplex. Females of *T. williami* (*n* = 5), were obtained from a laboratory colony. The females were cut transversely at the sixth abdominal segment and examined under scanning electron microscopy (SEM) at the Oswaldo Cruz/Fiocruz Institute Electronic Microscopy Platform.

**Results:**

It was possible to differentiate the three species based on the characteristics of urotergites VII, VIII and IX and urosternite VII, as well as the genital plaques, gonocoxites, and gonapophyses. To our knowledge, morphological differences in the spines present on gonapophysis 8 in triatomines are described here for the first time.

**Conclusions:**

The results show that external genitalia of females are useful structures to differentiate *T. costalimai*, *T. jatai* and *T. williami*. SEM analysis contributes to and corroborates, together with other tools morphological and molecular, the distinction of the three species.
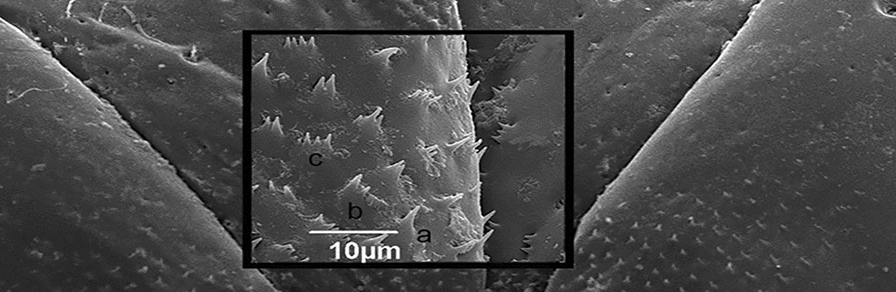

## Background

Triatomines are insect vectors of the *Trypanosoma cruzi* (Chagas, 1909), the causative agent of Chagas disease. It is estimated that about 6–8 million people are infected with *T. cruzi* worldwide, with the highest number of cases in Latin America [1]. According to data from the Brazilian Ministry of Health, there are approximately 12 million people with chronic Chagas disease in the Americas, with 2–3 million in Brazil [2]. The highest number of acute cases of the disease in Brazil from 2000 to 2017 was recorded in the northern region, where two of the species analyzed in this study, *Triatoma jatai* Gonçalves et al*.*, 2013 and *Triatoma costalimai* Verano & Galvão, 1959 are found. In this region, Chagas outbreaks from oral transmission highlight the importance of entomological surveillance.

Morphological studies generate important data that can be used in multidisciplinary approaches that answer questions about limits applicable to species, especially cryptic species or species with very similar morphology. The identification of triatomines is based on morphological characteristics [3, 4]. In the 1960s, a comparative analysis of the morphology of the external genitalia of both sexes by optical microscopy started to be used as an additional tool in the taxonomy of triatomines. However, the taxonomic value of the female genitalia morphology was questioned because it does not allow species identification in detail [5]. Since 2010, scanning electron microscopy (SEM) has been used for morphological characterization of the female genitalia of several triatomine species, proving to be an important complementary methodology for determining the taxonomy of the group [6]. The rupestrian species *T. jatai* and *T. costalimai* are distributed in the Cerrado biome, found in sympatry in the municipality of Paranã, Tocantins, Brazil, and show close similarities in terms of their external morphology and classical and geometric morphometrics [7]. Some studies also support the close genetic affinities between these two species [8, 9]. The description of *T. jatai* was made from specimens collected in the wild environment. Even though this species has been captured in an intradomestic environment, its vectorial capacity is still unknown and there is no record of natural infections with *T. cruzi* [10]. *Triatoma costalimai*, has a geographical distribution that covers the Brazilian states of Goiás, Tocantins, Minas Gerais, and Bahia, and has also been reported in Bolivia [4, 11]. This species has been found in wild, peridomestic and intradomestic environments, in some cases with a high prevalence of infection with *T. cruzi* [4, 10, 12, 13]. The rupestrian species *T. williami* Galvão, Souza & Lima, 1965, which is morphologically similar to *T. costalimai*, is found in the Cerrado Biome and Pantanal, in the states of Goiás, Mato Grosso do Sul and Mato Grosso [4]. The first record of a triatomine of this species with natural infection by *T. cruzi* was from Mato Grosso, from specimens captured in an intradomestic environment [14]. The three mentioned species, whose morphology of the female genitalia has not been studied so far, were included in Matogrossensis subcomplex [7, 15]. The present examined the external genitalia of *T. jatai*, *T. costalimai* and *T. williami* using scanning electron microscopy (SEM) with the aim to provide complementary data to the morphological characterization of these three species that could be useful for identification as well as to further integrative studies to allow a better species delimitation within the Matogrossensis subcomplex. Additionally, the fine morphological characterization of the female genitalia could be a source of putative new characters to be used in future phylogenetic studies to allow a better comprehension of the evolutionary history of this group of vectors.

## Methods

Specimens were captured by a team of the Interdisciplinary Entomological Surveillance Laboratory in Diptera and Hemiptera, Instituto Oswaldo Cruz/Fiocruz, Brazil, in the Brazilian municipalities of Paranã and Aurora do Tocantins, Tocantins, by active and passive search using animal bait traps [16] in 2011 (SISBIO License 43393). The specimens were identified using a dichotomous key for the Matogrossensis subcomplex [7] and kept at −20 °C in the laboratory, until now. Specimens of *T. williami*, which have a morphology close to that of *T. costalimai* [3], were provided by the National and International Reference Laboratory in Triatomine Taxonomy (LNIRTT/ IOC-Fiocruz). A total of 25 females of *T. jatai* (*n* = 10), *T. costalimai* (*n* = 10), and *T. williami* (*n* = 5) were analyzed. The triatomines were sectioned with a scalpel in the transverse direction at the height of the sixth abdominal segment. The terminal portion of the abdomen was washed, dehydrated in an alcohol solution by immersion for 10 min at a series of concentrations (7.5%, 15%, 30%, 50%, 70%, 90% and three times at 100%). The structures were mounted on a metallic support on double-sided adhesive tape in the ventral or dorsal positions, forming a 90° angle with the base of the support. Samples were then left in an oven (Lufeco, Germany) at 50 °C for drying for 24 h and in a desiccator containing silica gel until metallization [17]. Subsequently, they were metallized with gold and examined under a JSM 6390 LV (JEOL USA Inc., Peabody, MA, USA) scanning electron microscope at the Oswaldo Cruz/FIOCRUZ Institute Electronic Microscopy Platform.

## Results

**Fig. 1:**
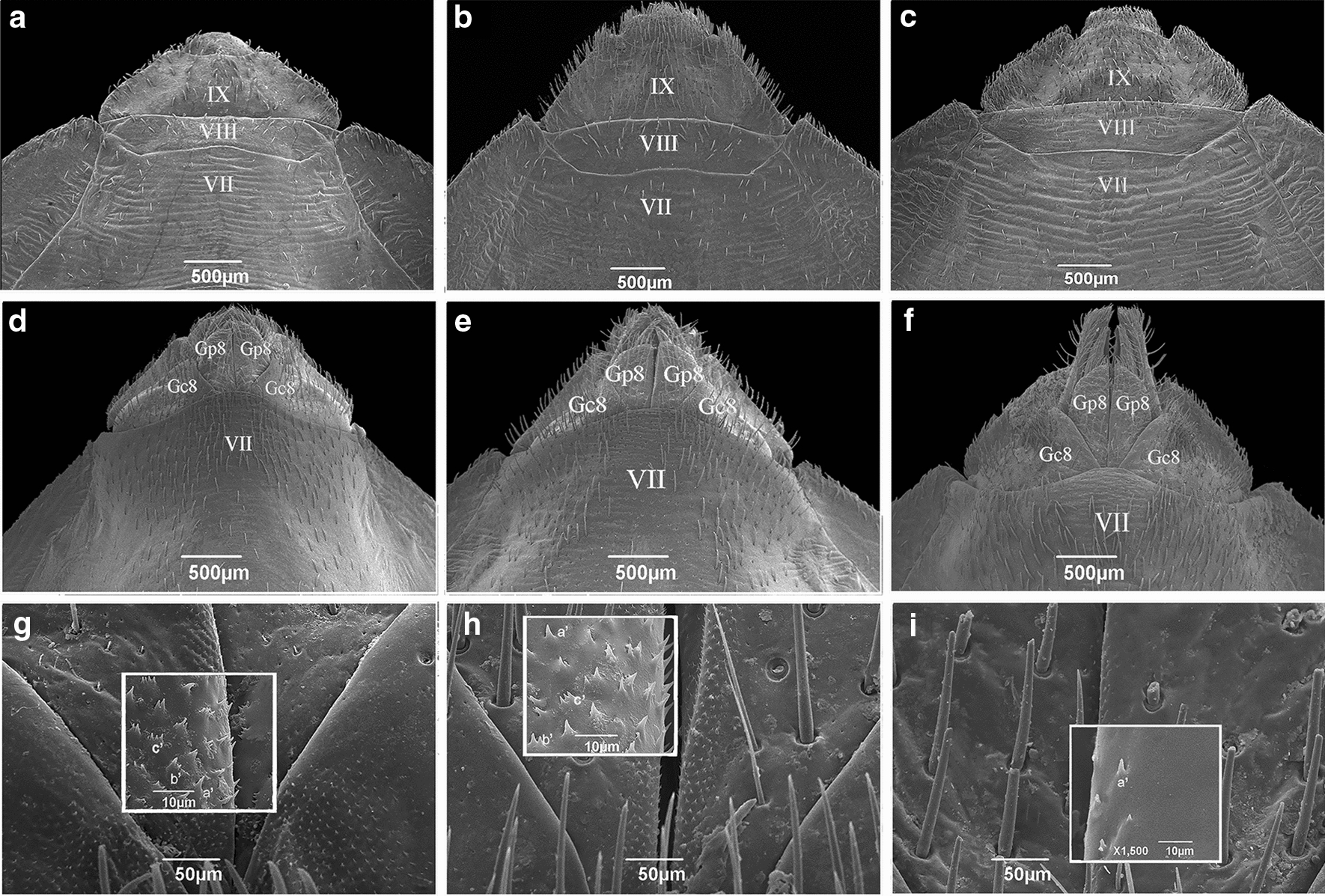
Electromicrographs of the external genitalia of female *Triatoma* spp. **a**–**c** Dorsal view. **a** *Triatoma jatai*. **b** *Triatoma costalimai*. **c** *Triatoma williami* (VII, 7th urotergite; VIII, 8th urotergite; IX, 9th urotergite). **d**–**f** Ventral view. **d** *Triatoma jatai*. **e** *Triatoma costalimai*. **f** *Triatoma williami* (VII, 7th urosternite; Gc8, gonocoxite; Gp8 - gonapophysis). **g** Median region of gonapophysis 8 (Gc8) in *Triatoma jatai* in detail: single-pointed (**a′**), bifurcated (**b′**) and trifurcated (**c′**); **h** *Triatoma costalimai*, in detail: single-pointed (**a′**), bifurcated (**b′**), and trifurcated (**c′**); **i** *Triatoma williami* in detail with single-pointed spines

Observation of the external genitalia of *T. jatai* females in dorsal view showed that the posterior edge of urotergite VII has a W-shape, with a 1 + 1 lateral depressions rising in the median region; the posterior edge of urotergite VIII is rectilinear, decaying laterally, and ending at half of the suture of the rounded apex connective; urotergite IX is trapezoidal, with bulging lateral walls and covered by thick, short, sparse setae that end near the anal tube (Fig. 1a).

*Triatoma costalimai* has a non-rectilinear posterior edge of urotergite VII, ending near the suture of the connective; the posterior edge of urotergite VIII is convex and ends at half of the suture of the connective with pointed apex; and urotergite IX is trapezoidal, with straight lateral walls and a 1 + 1 apical recesses near the anal tube, covered with thick, long setae (Fig. 1b).

In *T. williami*, the posterior edge of urotergite VII is rectilinear at the median region rising at the lateral margins towards the connexival suture; the posterior edge of urotergite VIII is rectilinear ending at the median line of the connexival suture; and urotergite IX is trapezoidal, with bulging lateral walls, presenting a 1 + 1 sharp depressions close to the anal tube, covered by short thick setae (Fig. 1c).

In *T. jatai*, ventrally, the line of the posterior edge of urosternite VIII is prominent in the median region, and the gonocoxites of the 8th segment (Gc8) are long and wide (Fig. 1d). In *T. costalimai*, the posterior edge line of urosternite VII is convex and Gc8 is long and narrow (Fig. 1e). In *T. williami*, the posterior edge of urosternite VII has the same shape as in *T. jatai*, but is not prominent. Gc8 is triangular and close to the median line of urosternite VII (Fig. 1f). In the median region of gonapophysis 8 (Gp8) and at the base of *T. jatai* Gc8, short spines were found on the apex with single, bifurcated, and trifurcated points (Fig. 1g). Similar spines were observed only in the median region of *T. costalimai* Gp8 (Fig. 1h). In *T. williami*, only short single-pointed apex spines were observed in the median region of Gp8 (Fig. 1i).

A summary of main morphological characteristics of the external female genitalia of *T. jatai*, *T. costalimai* and *T. williami* in dorsal and ventral view are show in Table 1.

**Table 1:** Main morphological characteristics of the external genitalia of *Triatoma jatai*, *Triatoma costalimai* and *Triatoma williami*

	Dorsal view, posterior edge			Ventral view		
	Urotergite VII	Urotergite VIII	Urotergite IX	Urosternite 8, posterior edge	Gonocoxites 8, shape	Gonapophysis 8, median region
*T. jatai*	W-shaped, with a 1 + 1 lateral depression rising in the median region (Fig. 1a)	Rectilinear, decaying laterally, ending at half of the suture of the rounded apex connective (Fig. 1a)	Trapezoidal, with bulging lateral walls covered by thick, short, sparse setae that end near the anal tube (Fig. 1a)	Prominent in the median region (Fig. 1d)	Long and wide (Fig. 1d)	Short spines on the apex and at the base with single, bifurcated, and trifurcated points (Fig. 1g)
*T. costalimai*	Non-rectilinear posterior edge, ending near the suture of the connective (Fig. 1b)	Convex and ends at half of the suture of the connective with poingted apex (Fig. 1b)	Trapezoidal, with straight lateral walls, 1 + 1 apical recesses near the anal tube, covered with thick, long setae (Fig. 1b)	Convex (Fig. 1e)	Long and narrow (Fig. 1e)	Short spines on the apex with single, bifurcated, and trifurcated points (Fig. 1h)
*T. williami*	Rectilinear at the median region rising at the lateral margins towards the connexival suture (Fig. 1c)	Rectilinear ending at the median line of the connexival suture (Fig. 1c)	Trapezoidal, with bulging lateral walls, presenting a 1 + 1 sharp depressions close to the anal tube, covered by short, thick setae (Fig. 1c)	Not prominent (Fig. 1f)	Triangular and close to the median line of urosternite VII (Fig. 1f)	Short single-pointed apex spines (Fig. 1i)

## Discussion

We conducted an exhaustive morphological analysis using scanning electron microscopy on the external female genitalia of *T. jatai*, *T. costalimai* and *T. williami*. Although the characterization of the external genitalia of females whose taxonomic value of optical microscopy was questioned [5], the results showed that scanning electronic microscopy is an efficient tool for differentiating *T. jatai*, *T. costalimai* and *T. williami*. Intraspecific polymorphism was not observed; however, the interspecific polymorphism is more expressed in urotergites and urosternites. The genital plates that allow the coupling of the genitalia show a difference in morphology, however, allow to speculate the possibility of crossing between *T. jatai* and *T. costalimai*, since they live in sympatry in the municipality of Paranã. Research along this line are being carried out in our laboratory to assess the possibility of crossbreeding and the formation of hybrids. This result will allow to associate the morphological aspect with the biology of reproduction. These results reinforce the differentiation of species and complement the multidisciplinary approach of using external morphology, classical and geometric morphometrics, and phylogenetic analyses, which led to the confirmation of the specific status of *T. jatai* [7–9]. Comparative morphology studies of triatomines, including similar species of the genera *Rhodnius*, *Triatoma*, *Panstrongylus* and *Meccus* [6, 18–23] have demonstrated the importance of this methodology as a complementary approach in relation to the method for identification of triatomines [3]. This approach was also used for cryptic species that are part of the Brasiliensis complex allowing differentiation between them [24].

The presence of single-pointed, bifurcated, and trifurcated spines in the median region of gonapophysis 8 in the three species studied, and at the base of gonocoxite 8 in *T. jatai*, shows the importance of the morphological details detectable by using SEM. These spines are present in the internal genitalia of *Rhodnius neglectus* (Lent, 1954), and also in the copulatory pouch, indicating that they may assist with spermatophore compression and sperm release [25]. However, the functions of these spines in copulation or ovulation have not been clarified. In a recent molecular phylogeny study [26], a reorganization of *Triatoma* groupings was suggested and left the classification of the sister species *T. jatai* and *T. costalimai* as undefined. However, they were included in the *T. pseudomaculata* species group, where *T. williami* is also found. Although both species occur in the Cerrado Biome, the difference in phytogeography and climate profile between the municipalities of Paranã and Aurora do Tocantins may be possible factors related to a recent process of speciation. In phenotypic terms, a difference has already been expressed in the morphology of the male genitalia [5] and now also for the female genitalia.

A high rate of natural infection of *T. costalimai* by *T. cruzi* in peridomestic and intradomestic environments has been reported [8, 10, 11]. Expanding understanding of the characteristics that differentiate related species is important for a more accurate diagnosis, and can facilitate entomological surveillance and control of vector transmission of Chagas disease, especially in municipalities in the south and southeast Tocantins region such as Paranã and Aurora do Tocantins.

## Conclusions

It was possible to differentiate the three species based on the characteristics of urotergites VII, VIII and IX and urosternite VII, as well as the genital plaques, gonocoxites, and gonapophyses.

The results contribute to and corroborate, together with other morphological and molecular studies, the differentiation of *T. jatai*, *T. costalimai* and *T. williami*, as well as the taxonomy of these species. It may also help to evaluate the reproductive compatibility between *T. jatai* and *T. costalimai* and the possible formation of hybrids under artificial conditions. These analyses are underway in order to fully contemplate all definitions of biological species.

## Data Availability

The datasets used and/or analyzed during the present study are available from the corresponding author upon reasonable request.

## Abbreviations

Fiocruz: Oswaldo Cruz Foundation
Gc 8: gonocoxite of segment VIII
Gp 8: gonapophysis of segment VIII
IOC: Oswaldo Cruz Institute
LNIRTT: National and International Reference Laboratory in Triatomine Taxonomy
MS: Ministry of Health
PAHO: Pan American Health Organization
SEM: scanning electron microscopy
SISBIO: Remote Attendance System of the Ministry of the Environment, Brazil
SVS: Health Surveillance Secretary
WHO: World Health Organization

## References

[1] PAHO/WHO. Chagas disease in the Americas: a review of the current public health situation and a vision for the future. Report: conclusions and recommendations. Washington: Pan American Health Organization and World Health Organization; 2018.

[2] SVS/MS (2019). Acute Chagas disease and spatial distribution of triatomines of epidemiological importance, Brazil, 2012 to 2016. Bol Epidemiol..

[3] Lent H, Wygodzinsky P (1979). Revision of the Triatominae (Hemiptera: Reduviidae), and their significance as vectors of Chagas disease. Bull Amer Mus Nat Hist..

[4] Galvão C, Dale C, Galvão C (2015). Identification keys for adults. Vectors of Chagas disease in Brazil. Zoological series: identification guides and manuals.

[5] Jurberg J, Lent H, Galvão C, Carcavallo RU, Galíndez Girón I, Jurberg J, Lent H (1997). The male genitalia and its importance in taxonomy. Atlas dos Vetores da Doença de Chagas nas Américas.

[6] Rosa JA, Mendonça VJ, Rocha CS, Gardim S, Cilense M (2010). Characterization of the external female genitalia of six species of Triatominae (Hemiptera: Reduviidae) by scanning electron microscopy. Mem Inst Oswaldo Cruz..

[7] Gonçalves TCM, Teves-Neves SC, Santos-Mallet JR, Carbajal-de-la-Fuente AL, Lopes CM (2013). *Triatoma jatai* sp. nov. in the state of Tocantins, Brazil (Hemiptera: Reduviidae: Triatominae). Mem Inst Oswaldo Cruz..

[8] Teves SC, Gardim S, de la Carbajal Fuente AL, Lopes CM, Gonçalves TCM, Santos-Mallet JR (2016). Mitochondrial genes reveal *Triatoma jatai* as a sister species to *Triatoma costalimai* (Reduviidae: Triatominae). Am J Trop Med Hyg..

[9] Pita S, Lorite P, Nattero J, Galvão C, Alevi KCC, Teves SC (2016). New arrangements on several species subcomplexes of *Triatoma* genus based on the chromosomal position of ribosomal genes (Hemiptera: Triatominae). Infect Genet Evol..

[10] Brito RN, Diotaiuti L, Gomes ACF, Souza RCM, Abad-Franch F (2017). *Triatoma costalimai* (Hemiptera: Reduviidae) in and around houses of Tocantins State, Brazil, 2005–2014. J Med Entomol..

[11] Justi SA, Russo CA, Santos-Mallet JR, Obara MT, Galvão C (2014). Molecular phylogeny of Triatomini (Hemiptera: Reduviidae: Triatominae). Parasit Vectors..

[12] Brito RN, Gorla DE, Diotaiuti L, Gomes ACF, Souza RCM, Abad-Franch F (2017). Drivers of house invasion by sylvatic Chagas disease vectors in the Amazon-Cerrado transition: a multi-year, state-wide assessment of municipality-aggregated surveillance data. PLoS Negl Trop Dis..

[13] Teves SC, Toma HK, Lopes CM, Oliveira BLN, Carbajal-de-la-Fuente AL, Souza DM (2019). *Triatoma costalimai* naturally infected by *Trypanosoma cruzi*: a public health concern. Am J Trop Med Hyg..

[14] Arraias-Silva WW, Rodrigues RSV, de Moraes LN, Venere PC, Lunardi RR, Souza IL, de Souto PCS (2011). First report of occurrence of Triatoma williami Galvão, Souza e Lima, 1965 naturally infected with *Trypanosoma cruzi* Chagas, 1909 in the State of Mato Grosso, Brazil. Asian Pacific J Trop Dis..

[15] Schofield CJ, Galvão C (2009). Classification, evolution and species groups within the Triatominae. Acta Trop..

[16] Noireau F, Flores R, Vargas F (1999). Trapping sylvatic Triatominae (Reduviidae) in hollow trees. Trans R Soc Trop Med Hyg..

[17] Santos-Mallet JR, Lopes CM, Gonçalves TCM, Ribeiro CAO, Reis-Filho HS, Goötzner SR (2012). Methods used in invertebrates. Técnicas e métodos para utilização prática em microscopia.

[18] Rosa JA, Rocha CS, Gardim S, Pinto MC, Mendonça VJ, Ferreira Filho JCR (2012). Description of *Rhodnius montenegrensis* n. sp. (Hemiptera: Reduviidae: Triatominae) from the state of Rondônia, Brazil. Zootaxa..

[19] Rosa JA, Mendonça VJ, Gardim S, Carvalho DB, Oliveira J, Nascimento JD (2014). Study of the external female genitalia of 14 *Rhodnius* species (Hemiptera, Reduviidae, Triatominae) using scanning electron microscopy. Parasit Vectors..

[20] Rosa JA, Justino HHG, Nascimento JD, Mendonça VJ, Rocha CS, Carvalho DB (2017). A new species of *Rhodnius* from Brazil (Hemiptera: Reduviidae: Triatominae). ZooKeys..

[21] Rivas N, Sánchez-Cordero V, Camacho AD, Alejandre-Aguilar R (2017). External female genitalia of six species od the genus *Meccus* (Hemiptera: Reduviidae: Triatominae). J Vector Ecol..

[22] Rodrigues JMDS, Rosa JA, Moreira FFF, Galvão C (2018). Morphology of the terminal abdominal segments in females of Triatominae (Insecta: Hemiptera: Reduviidae). Acta Trop..

[23] Souza ES, Von Artzingen NCB, Furtado MB, Oliveira J, Nascimento JD, Vendrami DP (2016). Description of *Rhodnius marabaensis* sp. n. (Hemiptera: Reduviidae: Triatominae) from Pará State, Brazil. ZooKeys..

[24] Oliveira J, Alevi KCC, Almeida CE, Mendonça VJ, Costa J, Rosa JA (2020). *Triatoma brasiliensis* species complex: characterization of the external female genitalia. J Vector Ecol..

[25] Lourenço AP, Santos-Mallet JR, Freitas SPC (2013). Anatomy of the spermatophore in triatomines (Hemiptera, Reduviidae, Triatominae) and its applications to the study of Chagas disease vector biology. Am J Trop Med Hyg..

[26] Monteiro FA, Wirauch C, Félix M, Lazoski C, Abad-Franch F (2018). Chapter five - evolution, systematics, and biogeography of the Triatominae, vectors of Chagas disease. Adv Parasitol..

